# Identification of signaling pathways modifying human dopaminergic neuron development using a pluripotent stem cell-based high-throughput screening automated system: purinergic pathways as a proof-of-principle

**DOI:** 10.3389/fphar.2023.1152180

**Published:** 2023-06-26

**Authors:** Claire Boissart, Marie Lasbareilles, Johana Tournois, Laure Chatrousse, Thifaine Poullion, Alexandra Benchoua

**Affiliations:** ^1^ CECS, I-STEM, AFM, Neuroplasticity and Therapeutics, Corbeil-Essonnes, France; ^2^ INSERM UMR 861, I-STEM, AFM, Corbeil-Essonnes, France; ^3^ UEVE UMR 861, I-STEM, AFM, Corbeil-Essonnes, France; ^4^ CECS, I-STEM, AFM, Research and Technological Innovation, High Throughput Screening Plateform, Corbeil-Essonnes, France

**Keywords:** Purines, Lesch–Nyhan disease, dopamine, pluripotent stem cells, high-throughput screening, drug discovery

## Abstract

**Introduction:** Alteration in the development, maturation, and projection of dopaminergic neurons has been proposed to be associated with several neurological and psychiatric disorders. Therefore, understanding the signals modulating the genesis of human dopaminergic neurons is crucial to elucidate disease etiology and develop effective countermeasures.

**Methods:** In this study, we developed a screening model using human pluripotent stem cells to identify the modulators of dopaminergic neuron genesis. We set up a differentiation protocol to obtained floorplate midbrain progenitors competent to produce dopaminergic neurons and seeded them in a 384-well screening plate in a fully automated manner.

**Results and Discussion:** These progenitors were treated with a collection of small molecules to identify the compounds increasing dopaminergic neuron production. As a proof-of-principle, we screened a library of compounds targeting purine- and adenosine-dependent pathways and identified an adenosine receptor 3 agonist as a candidate molecule to increase dopaminergic neuron production under physiological conditions and in cells invalidated for the HPRT1 gene. This screening model can provide important insights into the etiology of various diseases affecting the dopaminergic circuit development and plasticity and be used to identify therapeutic molecules for these diseases.

## Introduction

Midbrain dopaminergic (DA) neurons innervate several areas of the brain, including the pre-frontal cortex, limbic system, and basal ganglia. They play an important role in the control of multiple brain functions, including voluntary movement and various behavioral processes, such as mood, reward, addiction, and stress. Alteration in the development, maturation, and projection of DA neurons, which can occur from the early postnatal phase to adolescence, has been proposed to be associated with many neurological and psychiatric disorders (Robbins, 2003; Cai et al., 2021), such as schizophrenia (Howes and Kapur, 2009), bipolar disorders (Ashok et al., 2017), addiction (Hyman et al., 2006), depression (Grace, 2016), attention-deficit/hyperactivity disorders (Gizer et al., 2009), autism spectrum disorders (Hamilton et al., 2013; Paval, 2017), and compulsive disorders (Maia and Conceicao, 2018). Therefore, understanding the signals modulating the production of human DA neurons is crucial to elucidate the mechanisms of disease emergence and progression and determine countermeasures. Human pluripotent stem cells (hPSCs) represent a valuable model for the systematic and analytical assessment of the consequences of interacting with molecular pathways during the early events of DA neuron development, which is difficult to replicate *in vivo*. Knowledge of the developmental biology of midbrain DA neurons has laid the foundation for their derivation from hPSCs. The first key steps in the production of DA neurons are the early patterning events that lead to the formation of the midbrain floor plate from the embryonic neural tube and are controlled by the regionalizing factors, sonic hedgehog (SHH), *Wnt*, and fibroblast growth factor 8 (FGF-8). Early midbrain floor plate cells, characterized by the co-expression of forkhead box A2 (FOXA2) and orthodenticle homeobox 2 (OTX2), give rise to different populations of progenitors that migrate to their final location and produce different types of midbrain neurons, including DA neurons, interneurons, oculomotor neurons, and neurons of the red nucleus. Specification of progenitors restricted to producing DA neuron progenitors starts with the expression of the homeobox gene engrailed-1 (*EN-1*), which controls the DA neuron development program. Expression of Nurr1 and Pitx3 promotes further differentiation of DA progenitors into post-mitotic neurons. Finally, expression of the enzymes responsible for DA synthesis, tyrosine hydroxylase (TH), and aromatic L-amino acid decarboxylase (AADC), gives mature DA neurons their identity. Midbrain DA neurons are ultimately located in the substantia nigra pars compacta, which innervates the basal ganglia and the ventral tegmental area, which projects to the limbic system and pre-frontal cortex (Hegarty et al., 2013; Mesman and Smidt, 2020). Methods for the directed differentiation of PSCs into DA neurons are based on these developmental principles. Most methods rely on mimicking the differentiation conditions described *in vivo* in animal models using soluble morphogens and growth factors (Kirkeby et al., 2017; Parmar et al., 2020; Antonov and Novosadova, 2021). The method involves the sequential exposure of hPSCs to SHH, WNT, and FGF-8. However, as these factors are not specific to DA progenitors, they only dose-dependently instruct the cells to adopt a midbrain floor plate regional identity and usually lead to the production of a limited number of authentic DA neurons mixed with undifferentiated progenitors and other types of midbrain neurons. However, what may be considered drawbacks of current protocols actually offer the possibility of using these systems to explore the specific molecular pathways influencing DA progenitors and neuron genesis.

In this study, we developed a high-throughput screening system using hPSCs to assess the effects of cell signaling pathway alterations on the early events of DA neuron development. We first developed a protocol allowing the differentiation and cultivation of highly self-renewing midbrain floor plate progenitors that can be harvested to constitute frozen cell banks. After thawing, these progenitors were seeded in a 384-well plate in an automated manner and treated with small molecules targeting specific cell signaling pathways. As a proof-of-principle, we focused our interest on purine metabolism because of its role in brain development (Fumagalli et al., 2017). We screened a library of compounds targeting purine- and adenosine-dependent pathways and identified an adenosine receptor agonist as a candidate molecule. We evaluated the activity of this molecule under physiological conditions and in a model of hypoxanthine-guanine phosphoribosyltransferase (HGPRT) deficiency, associated with abnormal development of DA neurons (Lloyd et al., 1981; Ernst et al., 1996; Torres and Puig, 2015).

## Materials and methods

### Pluripotent stem cells

A total of three iPSC lines, reprogrammed from fibroblasts, were used in this study. The detailed information on all cell lines is presented in Supplementary Table S1. Somatic cell reprogramming was performed using the four human genes OCT4, SOX2, c-Myc, and KLF4 cloned into Sendai viruses (Invitrogen, Cergy-Pontoise, France), and pluripotency was characterized as described by Takahashi et al. (2007). In addition to iPSCs, a control hESC line, SA001 (Cellartis, Goteborg, Sweden), was used under the supervision of the French Bioethics Agency (Agreement number NOR AFSB 12002 43S). PSC lines were maintained in StemMACS iPS-Brew XF medium (Miltenyi Biotec, Bergisch Gladbach, Germany) supplemented with 1:100 penicillin/streptomycin (Gibco, Grand Island, NY, United States) and vitronectin (Invitrogen, Carlsbad, CA, United States) as a matrix. The medium was changed every alternate day, and the cells were manually passaged once a week. Cell cultures were incubated at 37°C in a humidified atmosphere containing 5% CO_2_.

### Differentiation protocol of midbrain progenitors

**FIGURE 1:**
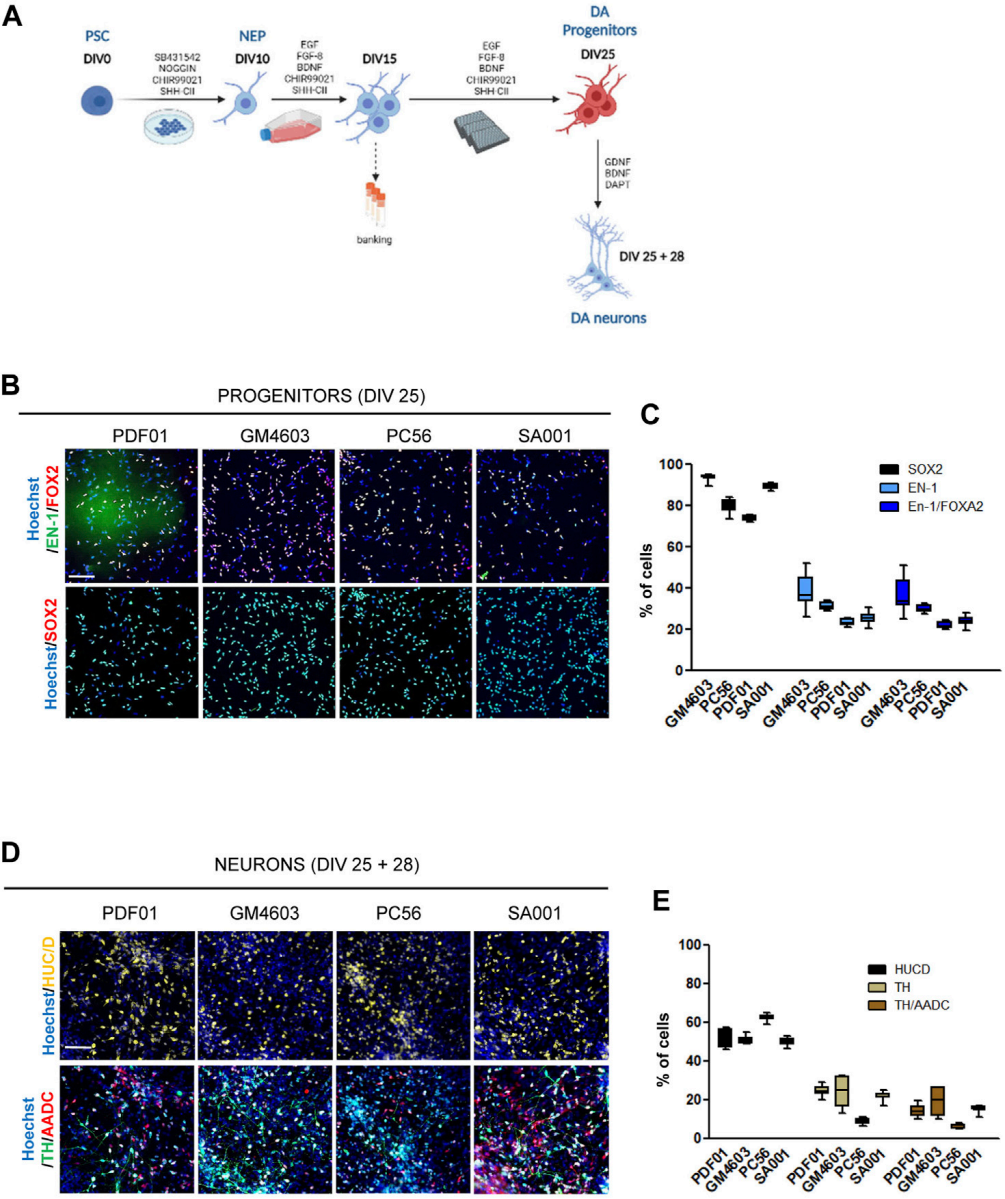
Differentiation of pluripotent stem cells into competent midbrain progenitors to produce dopaminergic neurons in a high-throughput screening format. **(A)** Schematic representation of the differentiation protocol. DIV, day *in vitro*; PSCs, pluripotent stem cells; NEPs, neuroepithelial cells; DA, dopamine. **(B)** Representative images of SRY-box transcription factor 2 (SOX2), engrailed homeobox 1 (EN-1), and forkhead box A2 (FOXA2) labeling of day 25 progenitors. Scale bar = 50 µm. **(C)** Quantification of the proportion of cells expressing the markers, SOX2, EN-1, and FOXA2, in a day 25 progenitor culture. Percentage of cells was calculated considering Hoechst-positive nuclei as the reference population. Box-and-whisker plots: boxes extend from 25th to 75th percentiles. Whiskers range from minimum to maximum values. Bars represent the median. N = 12 wells/condition. Each well represents independent differentiation. **(D)** Representative images of HuC/D, tyrosine hydroxylase (TH), and L-amino acid decarboxylase (AADC) labeling of neurons after 28 days of terminal differentiation of day 25 progenitors. Scale bar = 25 µm. **(E)** Quantification of the proportion of cells expressing the HuC/D, TH, and AADC markers in neuronal cultures. Percentage of cells was calculated considering Hoechst-positive nuclei as the reference population. Box-and-whisker plots: boxes extend from 25th to 75th percentiles. Whiskers range from minimum to maximum values. Bars represent the median. N = 8 wells/condition. Each well represents independent neuronal differentiation.

The PSC differentiation protocol for midbrain progenitors is shown in Figure 1. To initiate neural induction, PSCs were collected and embryonic bodies (EBs) were formed using low attachment 6-well plates in the N2B27 medium (DMEM/F12: Neurobasal [1:1], N2 supplement [1:100], and B27 supplement without vitamin A [1:50]) containing human recombinant Noggin (100 ng/mL; Peprotech, London, UK), SB431542 (10 μM; Tocris Biosciences, Ellisville, Missouri, United States), SHH-C24II (500 ng/mL, R&D System, Minneapolis, MN, United States), CHIR99021 (0.8 µM; Miltenyi Biotec.), and the rock inhibitor, Y27632 (10 ng/mL; Sigma-Aldrich, United States). The cell density on day 1 was 600,000 cells/well. The medium was replaced on day 2, and Y27632 was removed. After 4 days, EBs were seeded on culture dishes coated with poly-D-ornithine, laminin, and fibronectin (PoLF) in the same medium containing Y27632 at 10 ng/mL. On day 10, neural rosettes were manually collected under binocular observation and seeded in plastic wares coated with PoLF in the N2B27 medium containing the epidermal growth factor (EGF; 10 ng/mL; Peprotech), FGF-8 (100 ng/mL; Peprotech), brain-derived growth factor (BDNF; 20 ng/mL; Peprotech), CHIR99021 (0.8 µM), smoothened agonist (SAG; 500 nM; Millipore, Billerica, MA, United States), and ascorbic acid di-phosphate (AA2P; 0.2 mM; Sigma-Aldrich). This medium was considered a midbrain progenitor-amplifying medium. Y27632 was then added to the plate. The medium was changed every other day, and expanding progenitors were passaged at confluency using trypsin-EDTA in Y27632 containing the medium. On day 15, progenitors were frozen in dimethyl sulfoxide (DMSO)/fetal calf serum freezing medium (10/90%). On day 15, cells were seeded in PoLF-coated 384-well plates in a midbrain progenitor-amplifying medium at a density of 20,000 cells/cm^2^ until day 25, with medium changes every other day. Neuronal differentiation was started by plating day 25 progenitors at a density of 35,000 cells/cm^2^ in 384-well plates coated with PoLF in the N2B27 medium containing BDNF (20 ng/mL), glia-derived growth factor (GDNF; 10 ng/mL; Peprotech), and the ɣ-secretase inhibitor, N-[N-(3, 5-difluorophenacetyl)-l-alanyl]-S-phenylglycine t-butyl ester (DAPT; 1 μM; Tocris, Bristol, UK). The medium was changed twice a week.

### High-throughput drug screening assay and hit selection

To conduct high-throughput screening, day 15 progenitors were thawed and plated into PoLF-coated 384-well plates at 25,000 cells/cm^2^ in the progenitor-amplifying medium with Y27632 and without antibiotics using the Biocel 1800 liquid handling platform (Agilent Technologies, Santa Clara, CA, United States). Cells were treated with the chemical library, SCREEN-WELL Purinergic ligand library (Enzo Life Science, Villeurbanne, France; Supplementary Table S2). Compounds were added on days 16, 18, and 21 at 5 or 20 µM in quadruplicate. DMSO and H_2_O were used as the controls. On day 25, the cells were fixed with 4% paraformaldehyde (PFA) and processed for immunostaining.

### Immunostaining and high-content image analysis

Cells were fixed with 4% PFA (Electron Microscopy Sciences, Hatfield, PA, United States) and incubated overnight at 4°C with primary antibodies (Supplementary Table S3) diluted in a blocking buffer composed of 1x phosphate-buffered saline (PBS), 1% bovine serum albumin (BSA), and 0.3% Triton X-100 (Sigma-Aldrich). Cells were washed thrice with PBS and incubated for 45 min at room temperature with Alexa Fluor-conjugated secondary antibodies (Thermo Scientific, Les Ulis, France). Finally, the cells were washed thrice with PBS. In addition, Hoechst 33228 (1:3000; Sigma) nuclear staining was performed during secondary labeling. Automated, unbiased image acquisition and analysis were performed using a high-content analysis system, CellInsight HCS CX7, from Cellomics (Thermo Fisher Scientific). Cellular parameters were collected and quantified using the HCS Studio software.

### cAMP cellular content quantification

To measure the cellular content of cAMP following treatment with C08, day 16 progenitors were seeded in 96-well plates and treated for 1 h with 10 µM C08. Cells were lysed and cAMP quantified in the lysate using the CatchPoint Cyclic-AMP Fluorescent Assay kit following the manufacturer’s instructions (Molecular Devices, CA, United States).

### Compound selection and statistical analysis

For selection of hit compounds in the primary screening, the Z-score (Curtis et al., 2016) was calculated, which is defined as Z = (x−μ)/σ, where x is the percentage of EN-1/FOXA2 cells for an individual compound, μ is the mean percentage of EN-1/FOXA2 cells in all test compounds, and σ is the standard deviation of all test compounds. A compound with a minimum Z-score of 1.0 was considered a candidate compound.

For intergroup multiple comparisons during hit secondary screening and validation, PRISM software (GraphPad, San Diego, CA, United States) was used to analyze variance, followed by Bonferroni *post hoc* tests. Statistical significance was set at *p* < 0.01.

### Clustered regularly interspaced palindromic repeat (CRISPR)/CRISPR-associated protein 9 (Cas9) editing of HGPRT^–^/Y cells

*HGPRT* knockout cells were generated from the male hESC control line, SA001, via CRISPR/Cas9-mediated genome editing, as previously described (Ruillier et al., 2020). Protein knockout was regularly checked via Western blotting analysis using an HGPRT primary antibody and actin as a loading control (Supplementary Table S3).

## Results

### Differentiation of pluripotent stem cells into competent midbrain progenitors to produce DA neurons in a high-throughput screening manner

In this study, we aimed to construct a model for the development of hPSC-derived DA progenitors cultivable in a high-throughput miniaturized manner to simultaneously compare the effects of several experimental conditions. One of the key prerequisites was to obtain a robust and reproducible population of neural progenitors with the potential to produce DA neurons, which can be amplified to constitute frozen banks usable as a starting point to conduct high-throughput screening campaigns. To achieve this goal, we modified existing protocols to obtain a population of self-renewing progenitors that could maintain their competency to produce DA neurons in a dynamic range compatible with revealing compounds that increase or decrease the efficacy of conversion of these progenitors into the DA lineage. hPSCs were exposed to a cocktail of SMAD inhibitors to initiate neural differentiation, while the two regionalizing factors, SHH-C24II and CHIR99021, were used to promote ventral midbrain fates, as previously described (Kirkeby et al., 2012; Kirkeby et al., 2017). On day 10, we collected neural stem cells and further amplified them until day 15. The culture medium was modified accordingly to induce the proliferation of progenitors while maintaining their regional fate. We used a combination of EGF and FGF to promote progenitor self-renewal in a niche-independent manner (Conti et al., 2005; Boissart et al., 2013). FGF-8 was used instead of FGF-2 to support the caudal fates. SAG and CHIR were used to maintain the cellular fate. AA2P was added to support the metabolic requirements associated with intense self-renewal (Figure 1A). Progenitors were banked on day 15. After thawing, the progenitors were seeded in 384-well plates using a liquid-handling automated platform and cultivated until day 25 in the same medium. On day 25, progenitor fate was analyzed using HCA. In parallel, a fraction of these progenitors was terminally differentiated as post-mitotic neurons in a medium containing BDNF and GDNF for 28 d. The Notch inhibitor, DAPT, was added for the first 2 days (Figure 1A). This process was repeated simultaneously in four different hPSC lines (three induced pluripotent and one embryonic stem cell line; Supplementary Table S1). Progenitor cells were recovered optimally after thawing on day 15. After automated high-throughput seeding, they were homogeneously spread into the wells of the plate, allowing accurate evaluation of cell marker expression using HCA algorithms (Supplementary Figure S1). After 25 days of culture, the cell density was between 3000 and 4000 cells/well in a 384-well plate (Supplementary Figure S1). The majority of cells expressed the neural marker, SOX2, indicating homogenous engagement in the neural lineage (Figures 1B, C). Depending on the cell line, the percentage of cells expressing the DA progenitor marker, EN-1, was between 25% and 40%. Most EN-1 cells co-expressed the floor plate marker, FOXA2, confirming their presence in the midbrain floor plate region (Figures 1B, C). After 28 days of differentiation into a neuron-inducing medium, half of the population expressed the post-mitotic marker, HuC/D (Figures 1D, E). Neurons co-expressing TH and AADC were detected in cultures derived from all four cell lines, indicating the potential of EN-1 progenitors to produce mature DA neurons (Figures 1D, E). Together, we obtained a robust and reproducible protocol allowing progenitors to produce DA neurons, which can be used to further identify factors increasing the production of DA progenitors at the early stages.

### Primary screening of the compound library

**FIGURE 2:**
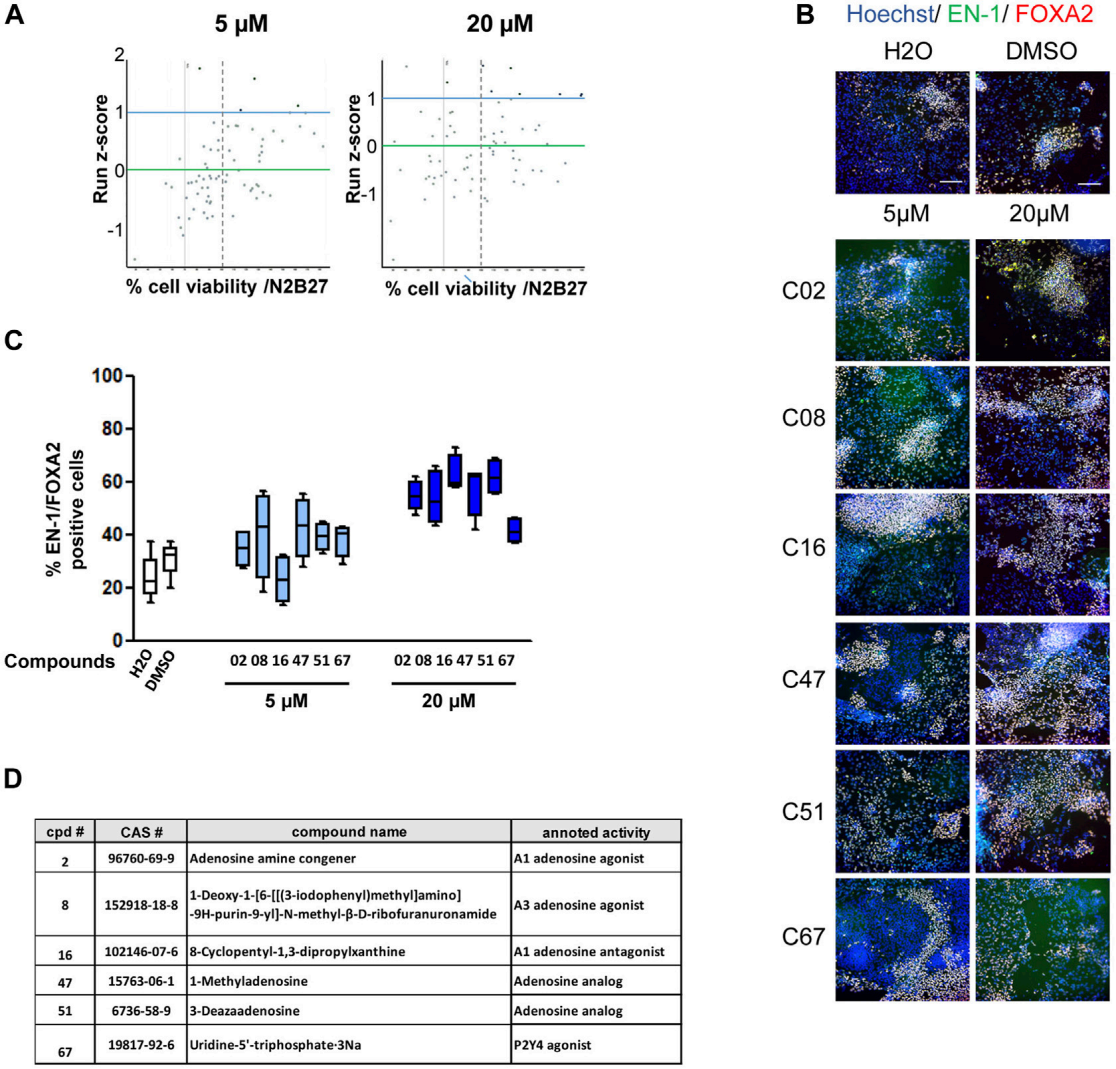
Primary screening of the purine and adenosine compound library. **(A)** Dot plot representation of screening results at 5 and 20 µM. Each dot represents the mean of the four wells treated with a given compound. Green horizontal bar represents the mean of all compounds tested (z-score = 0). Blue horizontal bar (z-score = 1) represents the limit above which a compound is considered a potential candidate. The vertical dashed line represents 70% cell survival compared to that in vehicle-treated cells. **(B)** Representative images of EN-1 and FOXA2 labeling of day 25 progenitors after treatment with the selected compounds. Scale bar = 50 µm. **(C)** Quantification of the proportion of cells expressing the markers, EN-1 and FOXA2, in day 25 progenitor cultures after treatment with the selected compounds. Percentage of cells was calculated considering Hoechst-positive nuclei as the reference population. Box-and-whisker plots: boxes extend from 25th to 75th percentiles. Whiskers range from minimum to maximum values. Bars represent the median. N = 4 wells/condition. **(D)** Selected compounds and their known reported activity.

Cultures of midbrain progenitors in 384-well plates were used to screen 73 compounds targeting purine- and adenosine-dependent pathways at high throughput and in an automated manner (Figure 2; Supplementary Table S2). Primary screening was performed using the PDF01 hiPSC line. Midbrain progenitors were seeded in 384-well plates on day 15. Cells were treated with compounds at 5 and 20 μM at each medium change between days 15 and 25 in quadruplicate (Figure 2A). On day 25, cells were fixed, and the expression levels of EN-1- and FOXA2-positive cells were quantified using automated high-content image-based analyses. Two criteria were used to select the hit compounds. First, cell viability, as assessed using Hoechst DNA labeling, should be above 70% of the cell viability measured in wells treated with vehicle only, that is, H_2_O or DMSO. Second, the raw quantification of EN-1/FOXA2 was converted into a run z-score to allow a standardized comparison of compounds between different plates (Curtis et al., 2016). We selected compounds with a run z-score >1, corresponding to a raw increase of more than 50% in the proportion of EN-1/FOXA2 cells compared with H_2_O or DMSO. Six compounds were selected at the end of the primary screening (Figure 2A). C08, C47, C51, and C67 were efficient at 5 μM, whereas C02 and C16 demonstrated efficiency only at 20 µM. For every compound, at the most efficient concentration, the percentage of EN-1/FOXA2 cells was above 45% of the total number of cells in the well. The percentage of EN-1/FOXA2 progenitors was between 23% and 30% in wells treated with H_2_O or DMSO, confirming an increase of at least 50% compared to that in the control conditions (Figures 2B, C). Annotated activities of these selected compounds indicated that adenosine-dependent pathways were mainly involved as C02-, C08-, and C16-targeted adenosine receptors, while C47 and C51 were described as adenosine analogs. The C67 compound acted as an agonist of the P2Y4 receptor (Figure 2D).

### Evaluation of the robustness and repeatability of the selected compound activity

**FIGURE 3:**
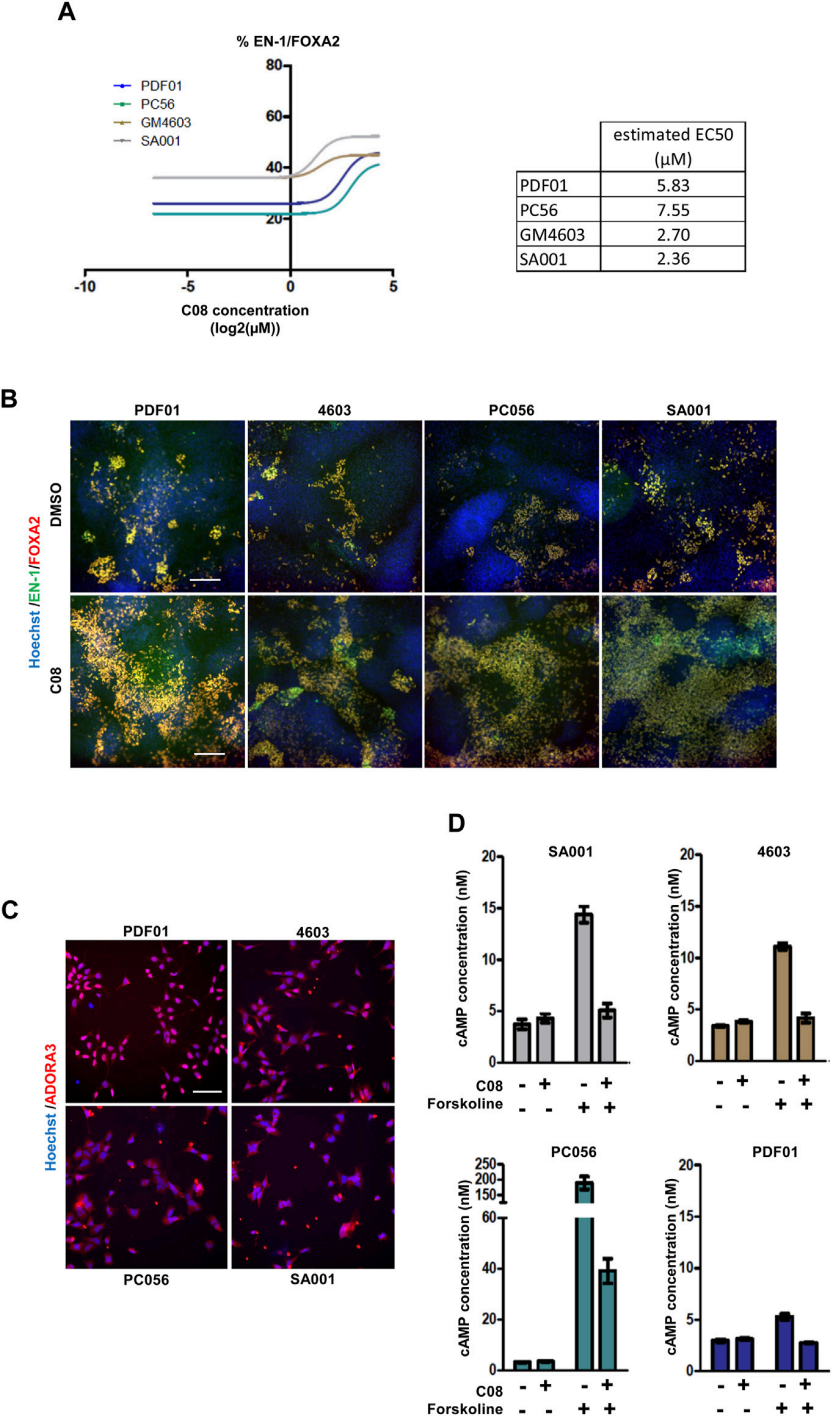
Evaluation of the robustness and repeatability of the selected compound activity. **(A)** Dose-dependent activity of C08 compounds. **(B)** Representative images of EN-1 and FOXA2 labeling in day 25 progenitors after treatment with C08 compounds. Scale bar = 50 µm. **(C)** Representative image of adenosine A3 receptor (ADORA3) labeling in day 16 progenitors. Scale bar = 25 µm. **(D)** Quantification of cAMP cellular content using ELISA following 1 h of exposure of day 16 progenitors with 10 µM C08. Bars represent mean +/- SD of three independent cultures per condition.

The six compounds were then tested in dose–response experiments to evaluate their repeatability across experiments. After retesting PDF01, each compound was also evaluated using the three other hPSC lines, PC56, GM4603, and SA001, to evaluate the robustness of their activity in different cell lines (Figure 3A; Supplementary Figure S1). Two compounds, C16 and C67, failed to demonstrate dose-dependent activity with PDF01. When robustness was evaluated by replicating the experiment on three other cell lines, three other compounds were excluded. C02, C47, and C51 did not demonstrate a robust effect across cell lines when the percentage of EN-1/FOXA2 cells was quantified. This retesting approach selects only one candidate compound that exhibits strong dose-dependent activity in every cell line tested. C08, an agonist of the adenosine A3 receptor (A3AR), was selected for further analysis (Figures 3A, B). We first confirmed that the A3AR protein, the physiological target of C08, was expressed in day 16 midbrain progenitors isolated from the four PSC lines (Figure 3C). In A3AR down-stream signaling, including Gi/cAMP-dependent and -independent pathways (Fishman et al., 2017), we measured cellular cAMP content following 1 h of exposure of progenitors to 10 µM C08 (Figure 3D). Treatment with C08 did not significantly reduce cAMP cellular content in progenitors cultivated in the amplification medium. In contrast, C08 was efficient at counteracting cAMP increase induced by forskolin, a direct activator of the adenylate cyclase, indicating that it was able to bind to A3AR and activate the Gi protein. In addition, phosphorylation of AKT, a downstream target of PKA, involved in the same cascade, was not induced (Supplementary Figure S3). Together, this indicated that, while able to bind to A3AR and activate Gi, C08 influenced DA genesis in our culture system in an adenylate cyclase/cAMP/Gi-independent manner.

### Evaluation of C08 activity under physiological conditions and in a HGPRT-deficiency model

**FIGURE 4:**
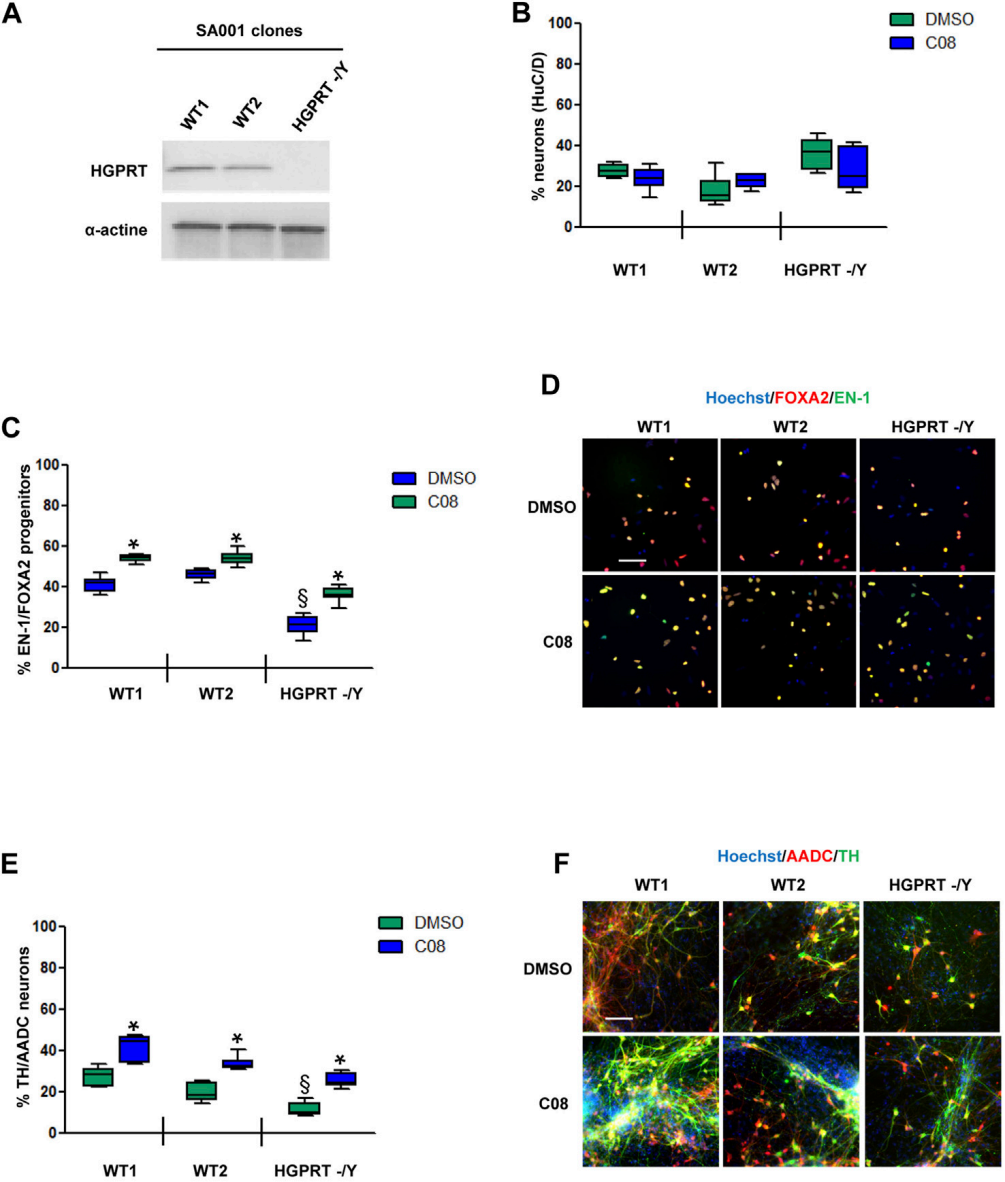
Evaluation of C08 activity in a model of HGPRT-deficiency **(A)** Western blotting analysis of HGPRT expression levels in SA001-derived clones. Actin was used as a loading control. **(B)** Quantification of the percentage of HuC/D-positive neurons produced by wild-type (WT) and HGPRT–/Y cells from day 25 progenitors, with or without C08 treatment. **(C)** Quantification of the proportion of cells expressing the markers, EN-1 and FOXA2, in day 25 progenitor cultures after treatment with C08. Box-and-whisker plots: boxes extend from 25th to 75th percentiles. Whiskers range from minimum to maximum values. Bars represent the median. N = 8 wells/condition. Each well represents an independent differentiation. **(D)** Representative images of EN-1 and FOXA2 labeling of day 25 progenitors after treatment with C08 compound. Scale bar = 25 µm. **(E)** Quantification of the proportion of neurons expressing the markers, TH and AADC, after 28 days of differentiation in a neuronal medium of 25 progenitor cultures treated with C08. Box-and-whisker plots: boxes extend from 25th to 75th percentiles. Whiskers range from minimum to maximum values. Bars represent the median. N = 6 wells/condition. Each well represents an independent differentiation. **(F)** Representative images of TH and AADC labeling of neurons after treatment with C08 compounds. Scale bars = 25 µm.

We then investigated C08 efficacy in increasing the genesis of DA progenitors and neurons under physiological conditions and in a disease model. We tested C08 in two clones of the WT hESC SA001 line and in one clone of SA001 invalidated for the *HGPRT* gene using CRISPR/Cas 9 (Ruillier et al., 2020) (Figure 4A). Compared to WT SA001 clones,HGPRT–/Y progenitors conserved their ability to produce post-mitoticneurons, as assessed using HuC/D pan-neuronal markers (Figure 4B). The three clones were differentiated side-by-side, and on day 25, the proportion of EN-1/FOXA2 progenitors was quantified. Loss of HGPRT activity was associated with a decreased number of EN-1-positive progenitors (Figures 4C, D). Day 25 progenitors were harvested and further differentiated into post-mitotic neurons for 28 d. Compared to WT SA001 clones, HGPRT^−^/Y progenitors conserved their ability to produce post-mitotic neurons, as assessed using HuC/D pan-neuronal markers, but quantification of the co-localized expression of TH and AADC enzymes in HuC/D-positive neurons indicated that HGPRT^−^/Y progenitors produced fewer DA neurons than their WT counterparts (Figures 4E, F; Supplementary Figure S4). The progenitors were then treated with 20 µM C08 at each medium change between days 15 and 25. Repeated treatment with C08 significantly increased the percentage of cells expressing EN-1/FOXA2 in WT and HGPRT^−^/Y cells (Bonferroni post-hoc tests, *p* < 0.01; Figures 4C, D). Along with significantly increasing the proportion of EN-1/FOXA2 progenitors, C08 also significantly increased the proportion of mature DA neurons in both WTs and HGPRT^−^/Y SA001 cultures. Interestingly, treatment with C08 was sufficient to correct DA neuron deficiency associated with HGPRT loss-of-function that was restored to the same level as that in WT cells in this cellular model (Bonferroni post-hoc tests *p* < 0.01; Figures 4E, F).

## Discussion

In this study, we developed a screening model based on iPSC-derived neural stem cells to identify modulators of human DA neuron differentiation. The screening cascade identified C08, also known as IB-MECA, an A3 adenosine receptor (A3AR) agonist, as a robust promoter of the very early stages of DA neuron genesis, both in physiological conditions and in neurons with HGPRT-deficiency as a model of Lesch–Nyhan disease (LND). To date, four types of adenosine receptors have been identified and characterized. These receptors are G protein-coupled receptors that modulate adenylyl cyclase-dependent signaling pathways. A3AR couples to Gi/Gq. After receptor binding to its agonists, the Gi protein inhibits adenylate cyclase, blocking the conversion of AMP into cAMP and inactivating protein kinase A (PKA). In addition, the Gq protein acts on signaling pathways regulated by phospholipase C, PI3 kinase, and MAP kinase. Thus, A3AR can modulate various physiological functions. In the adult brain, A3AR expression has been reported in the thalamus, hippocampus, cortex, and retinal ganglion cells, as well as in motor nerve terminals and pial and intercerebral arteries. A3ARs are also expressed in microglia and astrocytes (Borea et al., 2017). Pharmacological modulation of A3AR influences the evolution of brain damage following cerebral ischemia or traumatic injury (Melani et al., 2014; Bozdemir et al., 2021). A3AR is also involved in neuroinflammation (Farr et al., 2020) and pain control (Janes et al., 2016). To date, the expression of A3AR and its involvement in brain development are not well-documented. Our data suggest that A3AR-dependent pathways may be involved in DA neuron genesis through a cAMP-independent pathway. A3AR signals through both G protein-dependent (via Gi or β,γ subunits) and independent pathways ((Fishman et al., 2017)). Next to Gi, A3AR signals through Gq, activating PLC dependent pathways. Translocation of arrestin to the nucleus is associated with G protein-independent signaling, which includes direct mRNA transcription regulation ((DeWire et al., 2007)). Not all A3AR pathways are present in all cell types. The exact mechanisms of action of IB-MECA in our model need to be investigated further. Its specificity for binding to A3AR over other adenosine receptors also needs to be balanced.

In LND, the alteration of purine salvage pathways caused by the loss of function of the HGPRT enzyme deregulates genes involved in early neuronal development (Torres and Puig, 2015; Dinasarapu et al., 2022; Witteveen et al., 2022). We previously reported the therapeutic potential of adenosine-like molecules, particularly S-adenosyl methionine (SAMe), in an LND iPSC-derived neural stem cell model (Ruillier et al., 2020). We demonstrated that the protective effect of SAMe was accomplished via its conversion to AMP. The finding that IB-MECA corrected deficits associated with HGPRT deficiency in LND DA progenitors further supported the hypothesis that stimulating adenosine signaling represents an interesting target for the development of new treatments for LND. Several agonists of A3AR have been developed as druggable molecules, which may be repurposed for LND (Fishman, 2022). Interestingly, our screening model helped select small molecules, increasing the efficacy of the conversion of PSCs into DA progenitors, a strategy investigated for decades as a cell therapy product for Parkinson’s disease. Several protocols have been found in the literature describing the production of DA progenitors to replace lost neurons in patients with Parkinson’s disease that mainly use growth factor-based directed differentiation (Antonov and Novosadova, 2021). However, these protocols still demonstrated flaws, as they do not provide specificity for obtaining ventral midbrain DA neurons, as floor plate progenitors also generate other types of neurons. Consequently, the authors reported low DA neuron yields with high variability between cell lines and differentiation batches (Kee et al., 2017; Kirkeby et al., 2017; Kim et al., 2021). Therefore, our screening model can be used for the identification of small molecules that can enhance the amount and specificity of neurons produced and improvement of the standardization of differentiation protocols. Increasing the proportion of mature authentic DA neurons may enable drug screening for late neurodegenerative diseases, such as Parkinson’s disease and psychiatric disorders affecting the homeostasis of the DA circuit.

Our screening tool can be replicated using iPSCs from patients with defined clinical profiles, facilitating phenotypic disease modeling. Moreover, our tool can be used to identify therapeutic molecules for various diseases affecting DA circuit development and plasticity, which are agnostic to the molecular mechanisms involved in disease emergence and progression. This is of particular interest for rare or multifactorial diseases (Benchoua et al., 2021). It can also aid in the identification of disease-modifying pathways and genes at high throughput using CRISPR/CAS9-based whole-genome screening to provide insights into disease etiology (Nishiga et al., 2021). Together, our tool can aid in stratifying patients for personalized treatment and support the development of pharmacogenetics and pharmacogenomics to predict patient responses to drug therapy (Pardinas et al., 2021; Liu et al., 2022).

## Data Availability

The raw data supporting the conclusion of this article will be made available by the authors, without undue reservation.

## Supplementary material

SUPPLEMENTARY FIGURE S1 Seeding quality following automated cell dispensing. **(A)** Representative whole-well image of the cell dispersion of day 25 progenitors seeded on day 15 using automated liquid handling devices. Scale bar = 200 µm. **(B)** Quantification of the cell number per well. Each dot represents a single well. N = 12 wells per cell line. **(C)** Example of cell segmentation obtained after applying image analysis algorithms for quantification of different cell subtypes.

SUPPLEMENTARY FIGURE S2 Evaluation of the robustness and repeatability of the compound selected from primary screening.

SUPPLEMENTARY FIGURE S3 Phosphorylation of AKT at serine 473 following C08 treatment. Western-blot analyses of AKT and phospho-AKT content following 24 h of treatment of day 25 progenitors with C08. Beta-actin was used as a loading control.

SUPPLEMENTARY FIGURE S4 Dopamine release from PSC-derived DA neurons. ELISA quantification of DA in the supernatant of neuronal culture. DA concentration is expressed in ng/ml and normalized to the number of neurons present in the well measured using HUC/D labeling. Each dot represents a single well. N = 4 wells per cell line.

SUPPLEMENTARY TABLE S1 Pluripotent stem cell lines used in this study.

SUPPLEMENTARY TABLE S2 List and description of the compounds tested during the primary screening in this study.

SUPPLEMENTARY TABLE S3 List of the primary antibodies used in this study.

## References

[B1] Antonov S. A. Novosadova E. V. (2021). Current state-of-the-art and unresolved problems in using human induced pluripotent stem cell-derived dopamine neurons for Parkinson's disease drug development. Int. J. Mol. Sci. 22, 3381. 10.3390/ijms22073381 33806103 PMC8037675

[B2] Ashok A. H. Marques T. R. Jauhar S. Nour M. M. Goodwin G. M. Young A. H. (2017). The dopamine hypothesis of bipolar affective disorder: The state of the art and implications for treatment. Mol. psychiatry 22, 666–679. 10.1038/mp.2017.16 28289283 PMC5401767

[B3] Benchoua A. Lasbareilles M. Tournois J. (2021). Contribution of human pluripotent stem cell-based models to drug discovery for neurological disorders. Cells 10, 3290. 10.3390/cells10123290 34943799 PMC8699352

[B4] Boissart C. Poulet A. Georges P. Darville H. Julita E. Delorme R. (2013). Differentiation from human pluripotent stem cells of cortical neurons of the superficial layers amenable to psychiatric disease modeling and high-throughput drug screening. Transl. psychiatry 3, 294. 10.1038/tp.2013.71 PMC3756296 23962924

[B5] Borea P. A. Gessi S. Merighi S. Vincenzi F. Varani K. (2017). Pathological overproduction: The bad side of adenosine. Br. J. Pharmacol. 174, 1945–1960. 10.1111/bph.13763 28252203 PMC6398520

[B6] Bozdemir E. Vigil F. A. Chun S. H. Espinoza L. Bugay V. Khoury S. M. (2021). Neuroprotective roles of the adenosine A(3) receptor agonist AST-004 in mouse model of traumatic brain injury. Neurother. J. Am. Soc. Exp. Neurother. 18, 2707–2721. 10.1007/s13311-021-01113-7 PMC8804149 34608616

[B7] Cai Y. Xing L. Yang T. Chai R. Wang J. Bao J. (2021). The neurodevelopmental role of dopaminergic signaling in neurological disorders. Neurosci. Lett. 741, 135540. 10.1016/j.neulet.2020.135540 33278505

[B8] Conti L. Pollard S. M. Gorba T. Reitano E. Toselli M. Biella G. (2005). Niche-independent symmetrical self-renewal of a mammalian tissue stem cell. PLoS Biol. 3, 283. 10.1371/journal.pbio.0030283 PMC1184591 16086633

[B9] Curtis A. E. Smith T. A. Ziganshin B. A. Elefteriades J. A. (2016). The mystery of the Z-score. Aorta 4, 124–130. 10.12945/j.aorta.2016.16.014 28097194 PMC5217729

[B10] DeWire S. M. Ahn S. Lefkowitz R. J. Shenoy S. K. (2007). Beta-arrestins and cell signaling. Annu. Rev. physiology 69, 483–510. 10.1146/annurev.physiol.69.022405.154749 17305471

[B11] Dinasarapu A. R. Sutcliffe D. J. Seifar F. Visser J. E. Jinnah H. A. (2022). Abnormalities of neural stem cells in Lesch-Nyhan disease. J. neurogenetics 36, 81–87. 10.1080/01677063.2022.2129632 36226509 PMC9847586

[B12] Ernst M. Zametkin A. J. Matochik J. A. Pascualvaca D. Jons P. H. Hardy K. (1996). Presynaptic dopaminergic deficits in Lesch-Nyhan disease. N. Engl. J. Med. 334, 1568–1572. 10.1056/NEJM199606133342403 8628337

[B13] Farr S. A. Cuzzocrea S. Esposito E. Campolo M. Niehoff M. L. Doyle T. M. (2020). Adenosine A(3) receptor as a novel therapeutic target to reduce secondary events and improve neurocognitive functions following traumatic brain injury. J. neuroinflammation 17, 339. 10.1186/s12974-020-02009-7 33183330 PMC7659122

[B14] Fishman P. Bar‐Yehuda S. Liang B. T. Jacobson K. A. (2017). Pharmacological and therapeutic effects of A3 adenosine receptor agonists. Drug Discov. Today 17, 359–366. 10.1016/j.drudis.2011.10.007 PMC3289754 22033198

[B15] Fishman P. (2022). Drugs targeting the A3 adenosine receptor: Human clinical study data. Molecules 27, 3680. 10.3390/molecules27123680 35744805 PMC9229414

[B16] Fumagalli M. Lecca D. Abbracchio M. P. Ceruti S. (2017). Pathophysiological role of purines and pyrimidines in neurodevelopment: Unveiling new pharmacological approaches to congenital brain diseases. Front. Pharmacol. 8, 941. 10.3389/fphar.2017.00941 29375373 PMC5770749

[B17] Gizer I. R. Ficks C. Waldman I. D. (2009). Candidate gene studies of ADHD: A meta-analytic review. Hum. Genet. 126, 51–90. 10.1007/s00439-009-0694-x 19506906

[B18] Grace A. A. (2016). Dysregulation of the dopamine system in the pathophysiology of schizophrenia and depression. Nat. Rev. Neurosci. 17, 524–532. 10.1038/nrn.2016.57 27256556 PMC5166560

[B19] Hamilton P. J. Campbell N. G. Sharma S. Erreger K. Herborg Hansen F. Saunders C. (2013). De novo mutation in the dopamine transporter gene associates dopamine dysfunction with autism spectrum disorder. Mol. psychiatry 18, 1315–1323. 10.1038/mp.2013.102 23979605 PMC4046646

[B20] Hegarty S. V. Sullivan A. M. O'Keeffe G. W. (2013). Midbrain dopaminergic neurons: A review of the molecular circuitry that regulates their development. Dev. Biol. 379, 123–138. 10.1016/j.ydbio.2013.04.014 23603197

[B21] Howes O. D. Kapur S. (2009). The dopamine hypothesis of schizophrenia: Version III--the final common pathway. Schizophr. Bull. 35, 549–562. 10.1093/schbul/sbp006 19325164 PMC2669582

[B22] Hyman S. E. Malenka R. C. Nestler E. J. (2006). Neural mechanisms of addiction: The role of reward-related learning and memory. Annu. Rev. Neurosci. 29, 565–598. 10.1146/annurev.neuro.29.051605.113009 16776597

[B23] Janes K. Symons-Liguori A. M. Jacobson K. A. Salvemini D. (2016). Identification of A3 adenosine receptor agonists as novel non-narcotic analgesics. Br. J. Pharmacol. 173, 1253–1267. 10.1111/bph.13446 26804983 PMC4940822

[B24] Kee N. Volakakis N. Kirkeby A. Dahl L. Storvall H. Nolbrant S. (2017). Single-cell analysis reveals a close relationship between differentiating dopamine and subthalamic nucleus neuronal lineages. Cell. stem Cell. 20, 29–40. 10.1016/j.stem.2016.10.003 28094018

[B25] Kim T. W. Piao J. Koo S. Y. Kriks S. Chung S. Y. Betel D. (2021). Biphasic activation of WNT signaling facilitates the derivation of midbrain dopamine neurons from hESCs for translational use. Cell. stem Cell. 28, 343–355 e5. 10.1016/j.stem.2021.01.005 33545081 PMC8006469

[B26] Kirkeby A. Nelander J. Parmar M. (2012). Generating regionalized neuronal cells from pluripotency, a step-by-step protocol. Front. Cell. Neurosci. 6, 64. 10.3389/fncel.2012.00064 23316134 PMC3539732

[B27] Kirkeby A. Nolbrant S. Tiklova K. Heuer A. Kee N. Cardoso T. (2017). Predictive markers guide differentiation to improve graft outcome in clinical translation of hESC-based therapy for Parkinson's disease. Cell. stem Cell. 20, 135–148. 10.1016/j.stem.2016.09.004 28094017 PMC5222722

[B28] Liu J. S. Chen Y. Shi D. D. Zhang B. R. Pu J. L. (2022). Pharmacogenomics-a new frontier for individualized treatment of Parkinson's disease. Curr. Neuropharmacol. 21, 536–546. 10.2174/1570159X21666221229154830 PMC10207905 36582064

[B29] Lloyd K. G. Hornykiewicz O. Davidson L. Shannak K. Farley I. Goldstein M. (1981). Biochemical evidence of dysfunction of brain neurotransmitters in the Lesch-Nyhan syndrome. N. Engl. J. Med. 305, 1106–1111. 10.1056/NEJM198111053051902 6117011

[B30] Maia T. V. Conceicao V. A. (2018). Dopaminergic disturbances in tourette syndrome: An integrative account. Biol. psychiatry 84, 332–344. 10.1016/j.biopsych.2018.02.1172 29656800

[B31] Melani A. Pugliese A. M. Pedata F. (2014). Adenosine receptors in cerebral ischemia. Int. Rev. Neurobiol. 119, 309–348. 10.1016/B978-0-12-801022-8.00013-1 25175971

[B32] Mesman S. Smidt M. P. (2020). Acquisition of the midbrain dopaminergic neuronal identity. Int. J. Mol. Sci. 21, 4638. 10.3390/ijms21134638 32629812 PMC7369932

[B33] Nishiga M. Qi L. S. Wu J. C. (2021). CRISPRi/a screening with human iPSCs. Methods Mol. Biol. 2320, 261–281. 10.1007/978-1-0716-1484-6_23 34302664 PMC11047756

[B34] Pardinas A. F. Owen M. J. Walters J. T. R. (2021). Pharmacogenomics: A road ahead for precision medicine in psychiatry. Neuron 109, 3914–3929. 10.1016/j.neuron.2021.09.011 34619094

[B35] Parmar M. Grealish S. Henchcliffe C. (2020). The future of stem cell therapies for Parkinson disease. Nat. Rev. Neurosci. 21, 103–115. 10.1038/s41583-019-0257-7 31907406

[B36] Paval D. (2017). A dopamine hypothesis of autism spectrum disorder. Dev. Neurosci. 39, 355–360. 10.1159/000478725 28750400

[B37] Robbins T. W. (2003). Dopamine and cognition. Curr. Opin. neurology 16 (2), S1–S2. 10.1097/00019052-200312002-00001 15129843

[B38] Ruillier V. Tournois J. Boissart C. Lasbareilles M. Mahe G. Chatrousse L. (2020). Rescuing compounds for Lesch-Nyhan disease identified using stem cell-based phenotypic screening. JCI insight 5, 132094. 10.1172/jci.insight.132094 31990683 PMC7101145

[B39] Takahashi K. Tanabe K. Ohnuki M. Narita M. Ichisaka T. Tomoda K. (2007). Induction of pluripotent stem cells from adult human fibroblasts by defined factors. Cell. 131, 861–872. 10.1016/j.cell.2007.11.019 18035408

[B40] Torres R. J. Puig J. G. (2015). Hypoxanthine deregulates genes involved in early neuronal development. Implications in Lesch-Nyhan disease pathogenesis. J. Inherit. metabolic Dis. 38, 1109–1118. 10.1007/s10545-015-9854-4 25940910

[B41] Witteveen J. S. Loopstok S. R. Ballesteros L. L. Boonstra A. van Bakel N. H. M. van Boekel W. H. P. (2022). HGprt deficiency disrupts dopaminergic circuit development in a genetic mouse model of Lesch-Nyhan disease. Cell. Mol. life Sci. CMLS 79, 341. 10.1007/s00018-022-04326-x 35660973 PMC9167210

