# Supplementary figures and images for: Identification of signaling pathways modifying human dopaminergic neuron development using a pluripotent stem cell-based high-throughput screening automated system: purinergic pathways as a proof-of-principle
Source: Front Pharmacol. 2023 Jun 26;14:1152180. doi: 10.3389/fphar.2023.1152180 (PMC10331426; doi:10.3389/fphar.2023.1152180)

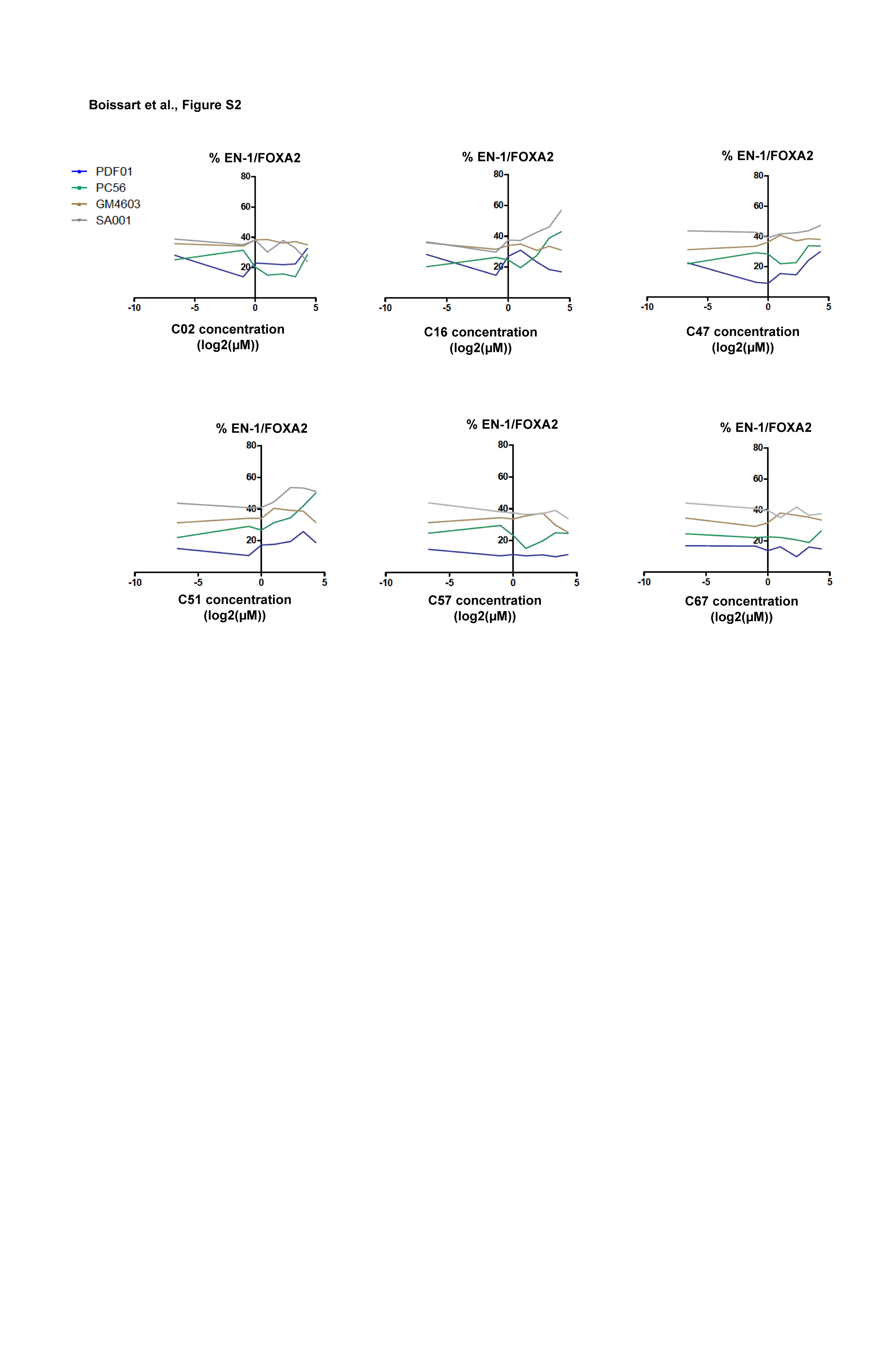

Supplement: Supplementary file 1 [file Image3.JPEG]

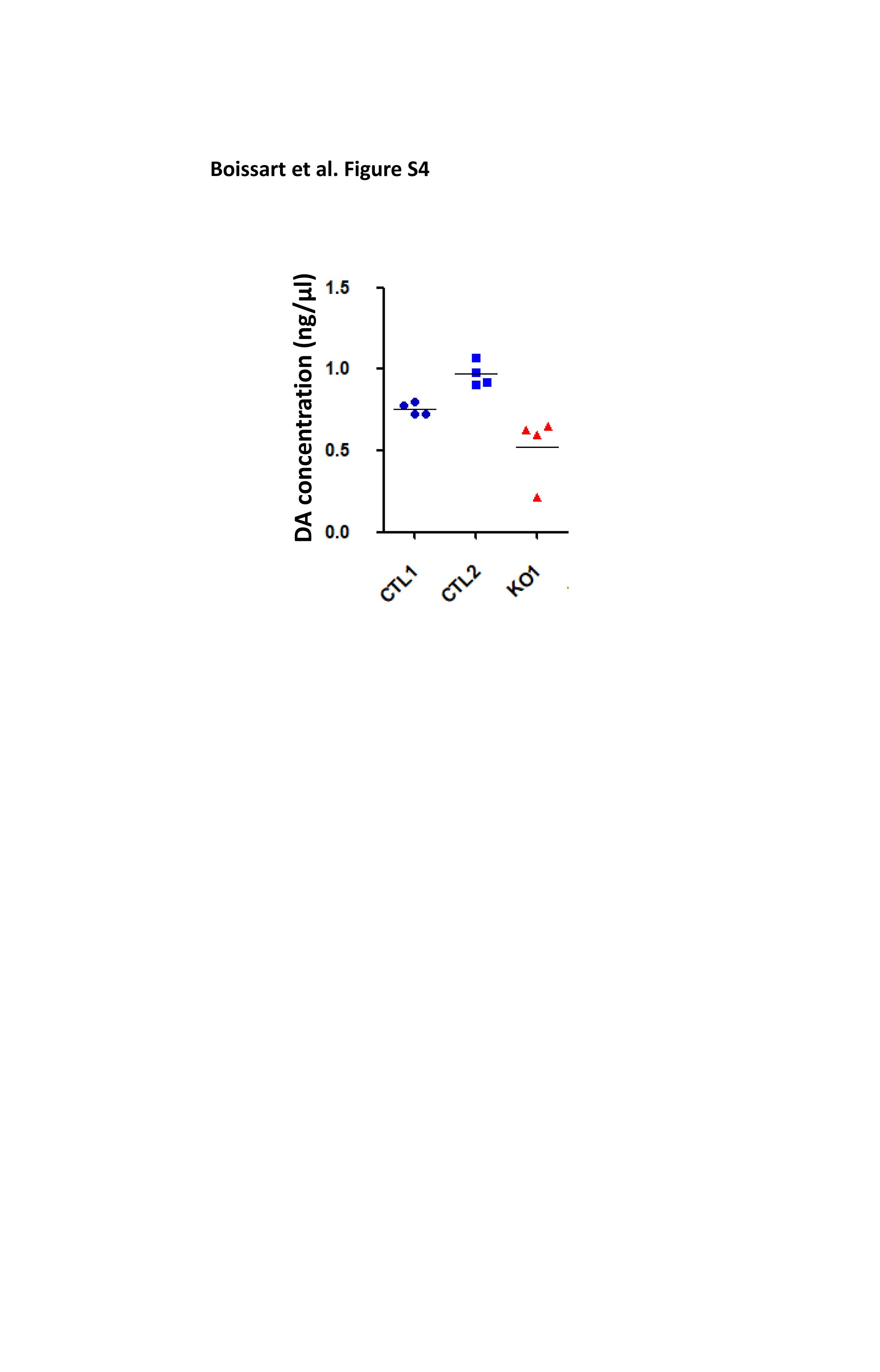

Supplement: Supplementary file 2 [file Image1.JPEG]

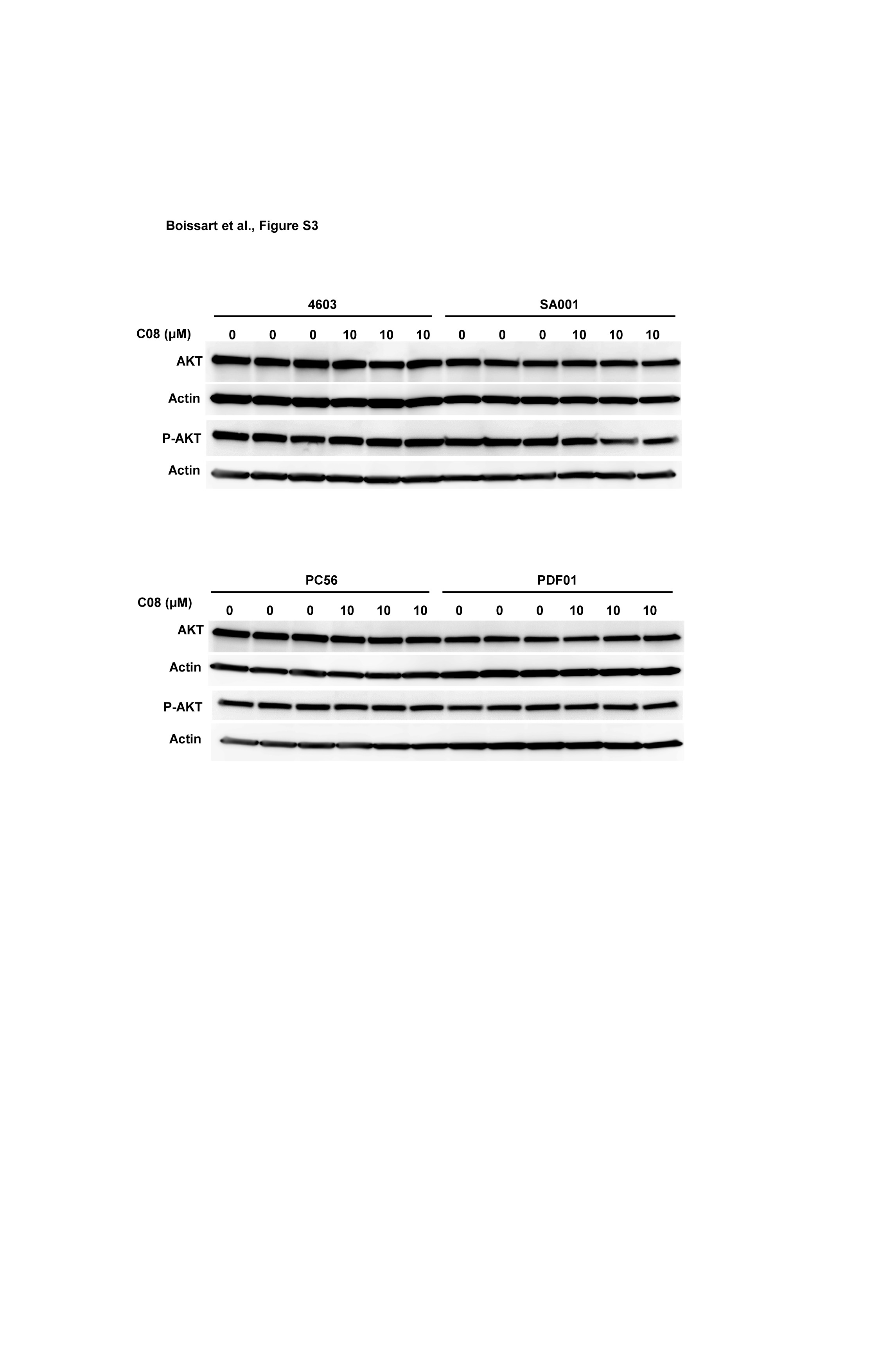

Supplement: Supplementary file 3 [file Image4.JPEG]

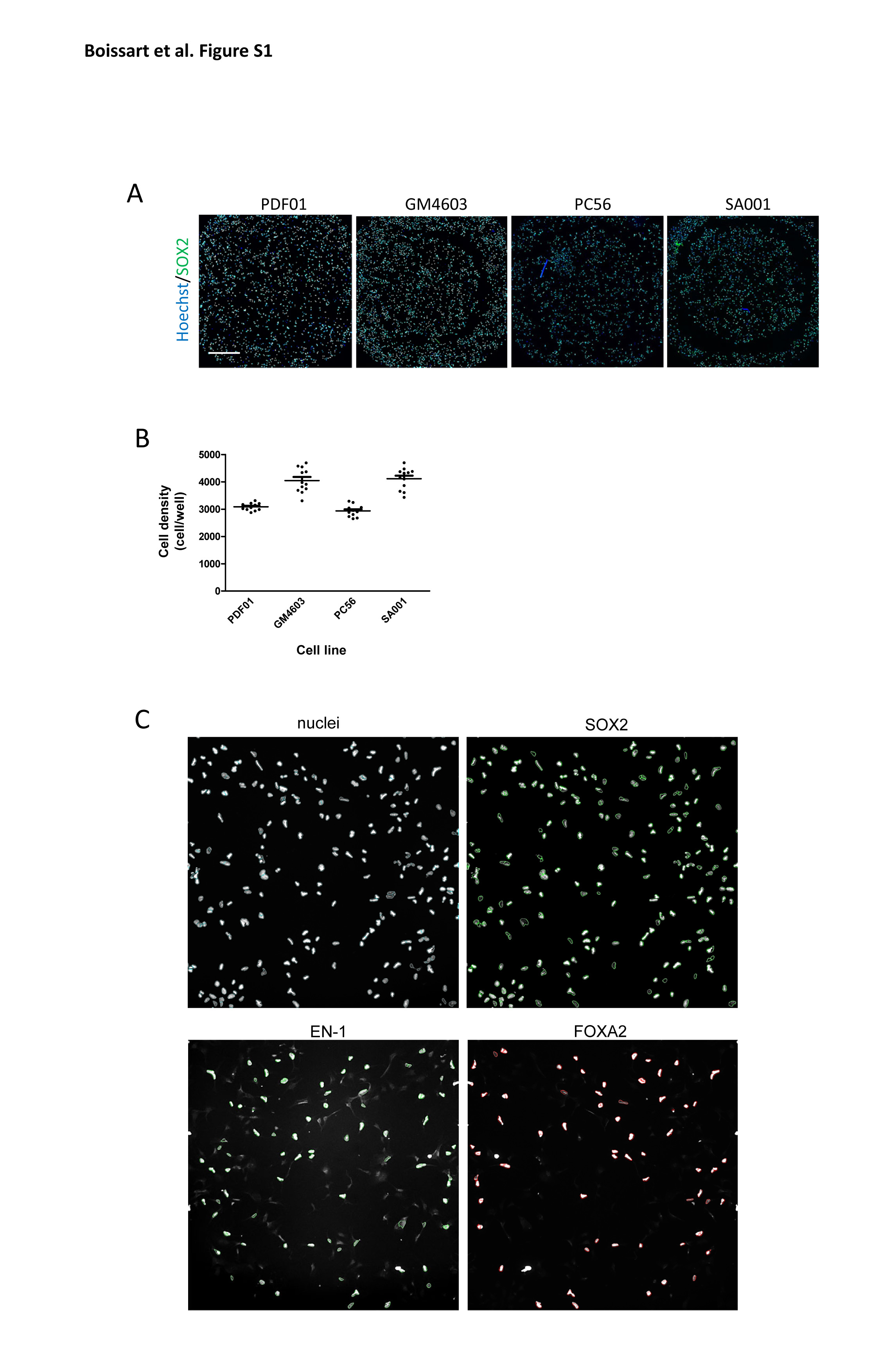

Supplement: Supplementary file 4 [file Image2.JPEG]
