# Supplementary material for: Identification of signaling pathways modifying human dopaminergic neuron development using a pluripotent stem cell-based high-throughput screening automated system: purinergic pathways as a proof-of-principle
Source: Front Pharmacol. 2023 Jun 26;14:1152180. doi: 10.3389/fphar.2023.1152180 (PMC10331426; doi:10.3389/fphar.2023.1152180)
Supplement: Supplementary file 5 [file Table2.DOCX]

| Target | Host | Reference | Provider | Dilution |
| --- | --- | --- | --- | --- |
| SOX2 | Rabbit | AB5603 | EMD Millipore Corp., Merck, Germany | 1 :500 |
| Engrail-1 (EN-1) | Mouse | 4G11 | DSHB, USA | 1 :100 |
| FOXA2 | Goat | AF2400 | R&D Systems®, USA | 1 :500 |
| Tyrosine Hydroxylase | Rabbit | ab112 | abcam, UK | 1 :500 |
| AADC | Goat | AF3564 | R&D Systems®, USA | 1 :100 |
| HuC/D neuronal protein | Mouse | A21271 | Invitrogen™ Thermo Scientific, USA | 1 :250 |
| ADORA3 | Rabbit | PA5-102048 | Invitrogen™ Thermo Scientific, USA | 1 :250 |
| HGPRT | Rabbit | 15059-1-AP | Proteintech, USA | 1 :1000 |
| Beta- Actin | Mouse | A3854 | Sigma-Aldrich, USA | 1 :50000 |
| AKT | Rabbit | 88800 | Cell signaling Technology,USA | 1 :1000 |
| Phospho-AKT (Ser 473) | Rabbit | 31957 | Cell signaling Technology,USA | 1 :1000 |

Lasbareilles et al. Table S3 : Antibodies

Table S3: List of the primary antibodies used in this study.
