# Supplementary material for: Identification of signaling pathways modifying human dopaminergic neuron development using a pluripotent stem cell-based high-throughput screening automated system: purinergic pathways as a proof-of-principle
Source: Front Pharmacol. 2023 Jun 26;14:1152180. doi: 10.3389/fphar.2023.1152180 (PMC10331426; doi:10.3389/fphar.2023.1152180)
Supplement: Supplementary file 6 [file Table3.DOCX]

| Cell type | Donnor code | Cell of origin | Provider | Karyotype |
| --- | --- | --- | --- | --- |
| hiPSC | PC56 | fibroblasts | Phenocell, France | 46, XX |
| hiPSC | GM04603 | fibroblasts | Coriell, USA | 46, XY |
| hiPSC | PDF01 | fibroblasts | EFS, France | 46, XX |
| hESC | SA001 | IVF embryo | Cellartis, sweden | 46, XY |

Lasbareilles et al Table S1
